# Research advances in early diagnosis and treatment strategies for peri-implantitis: from microecology to regenerative therapy

**DOI:** 10.3389/fcimb.2026.1863773

**Published:** 2026-08-05

**Authors:** Xuezhen Gao, Yuanna Zheng

**Affiliations:** 1 Department of Dentistry, Zhejiang Chinese Medical University, Hangzhou, Zhejiang, China; 2 Ningbo Dental Hospital/Ningbo Oral Health Research Institute, Ningbo, Zhejiang, China

**Keywords:** biomarkers, early diagnosis, guided bone regeneration, microbial dysbiosis, peri-implantitis

## Abstract

Peri-implantitis, a plaque-associated inflammatory disease characterized by progressive bone loss around functional implants, poses a significant threat to the long-term success of oral rehabilitation. The understanding of its etiology has evolved from a traditional infection model to a dysbiosis-based host-microbe interaction disorder model. This paradigm shift underscores the critical need for early detection and novel therapeutic strategies. This review comprehensively summarizes recent research advances within a logical framework: microbial dysbiosis, early diagnosis, treatment evolution, and regeneration. We detail the transition from a symbiotic microflora to a pathogenic biofilm, emphasizing key pathogens and host immune dysregulation mechanisms. The review evaluates the refinement of clinical and imaging diagnostics, the application of molecular biomarkers for early warning, and the emergence of point-of-care testing (POCT). Treatment strategies are discussed, moving beyond mechanical debridement to include innovative approaches like microecological modulation and regenerative therapies employing guided bone regeneration (GBR), bioactive factors, and tissue engineering concepts. Finally, we address current challenges in clinical translation and highlight future directions, including integrated diagnostics, personalized minimally invasive treatment, and smart biomaterials. The effective management of peri-implantitis necessitates an integrated strategy combining microbiological control with predictable tissue regeneration, requiring interdisciplinary collaboration to ensure the long-term stability of implant restorations.

## 1 Introduction

Over the past few decades, oral implant restoration technology has undergone substantial advancements, establishing itself as the gold standard for restoring masticatory function, aesthetic appearance, and quality of life in edentulous or partially edentulous patients (Reissmann et al., 2017; Homagarani et al., 2025). However, with the sharp increase in the global use of dental implants and the extension of their long-term survival, late-stage complications, particularly peri-implantitis, are increasingly becoming a prominent public health concern. Peri-implantitis is a plaque-associated pathological condition affecting the tissues surrounding an osseointegrated and functionally loaded dental implant, characterized by inflammation of the soft tissues and progressive loss of supporting bone (Berglundh et al., 2018a; Berglundh et al., 2018b).

Epidemiological studies highlight the clinical impact of this condition. A meta-analysis involving a large patient cohort revealed that the prevalence of peri-implantitis is approximately 19.83% at the patient level and around 9.25% at the implant level, with these figures showing an upward trend as the duration of implant service increases (Lee et al., 2017). This highly prevalent inflammatory condition can not only lead to increased implant mobility and eventual loosening or loss, resulting in prosthetic failure, but also impose the burden of frequent medical visits and substantial financial costs on patients. Therefore, timely and effective intervention for peri-implantitis to halt or slow disease progression is essential to ensure the long-term success of implant-supported restorations.

However, current clinical practice continues to face several diagnostic and therapeutic challenges. First, in terms of diagnosis, traditional clinical indicators—such as probing pocket depth (PPD), bleeding on probing (BOP), and two-dimensional radiographs—are inherently retrospective, reflecting tissue damage that has already occurred. By the time clinicians diagnose peri-implantitis using these methods, irreversible bone loss has often already taken place, resulting in missed opportunities for optimal, minimally invasive intervention (Heitz-Mayfield and Salvi, 2018a; Heitz-MayfieldSalvi, 2018b). Second, in terms of treatment, traditional non-surgical and surgical debridement methods, although effective in achieving short-term inflammatory control, often fail to ensure complete biofilm removal and thorough surface decontamination due to the implant’s complex macrostructural (e.g., threads) and microstructural (e.g., rough surface) features. This limitation contributes to persistently high recurrence rates (Figuero et al., 2014; Herrera et al., 2023).

In response to these challenges, the research paradigm in the scientific community is undergoing a profound shift—moving from a focus on end-stage disease management toward the investigation of initiating factors and early pathological processes. Accumulating evidence indicates that peri-implantitis results predominantly from an imbalance in the implant–microbiota–host triad, with microbial dysbiosis as its central driver (Hajishengallis, 2015). This understanding has established a theoretical foundation for the development of earlier and more accurate diagnostic methods as well as more targeted therapeutic strategies.

Based on this, the present review comprehensively synthesizes and critically evaluates key research advances in the field along a logically coherent framework: “microbial ecological imbalance (etiological basis) – early precise diagnosis (detection and early warning) – evolution of treatment strategies (infection control and immunomodulation) – regeneration and repair (functional reconstruction).” It further explores the intrinsic connections among these stages and outlines future research directions. The review aims to establish a comprehensive knowledge framework for clinicians—from etiology to tissue repair—and to provide valuable insights and references for related scientific investigations.

## 2 Microbiological basis and pathological mechanisms of peri-implantitis

Elucidating the microscopic transition from physiological homeostasis to pathological states is a foundational requirement for enabling early disease detection and informing the rational design of targeted therapeutic interventions. Central to this transition is the dysregulation of the dynamic equilibrium between the peri-implant microbiome and the host immune system.

### 2.1 Microecological characteristics of healthy peri-implant tissues

A functionally sound implant is closely surrounded by keratinized epithelial tissue at its transmucosal portion, forming a “peri-implant pocket” akin to the gingival sulcus of a natural tooth. This unique microenvironment provides a site for microbial colonization. Under healthy conditions, the microbial community in this region exhibits high diversity and stability, predominantly consisting of Gram-positive cocci and bacilli, such as *Streptococcus*, *Actinomyces*, and *Veillonella* (Chaturvedi et al., 2024; Chaturvedi et al., 2025). These early colonizing bacteria establish a “symbiotic” relationship with the host immune system. They occupy ecological niches, suppress the invasion of foreign pathogens, and maintain their metabolic byproducts at a low, controlled physiological inflammatory level when stimulating the host immune response. Key factors influencing microbial colonization include the patient’s oral hygiene status, salivary flow rate and composition, as well as the surface material and roughness of the implant/abutment (for example, sandblasted, large-grit, acid-etched [SLA] surfaces tend to facilitate more plaque accumulation compared to machined surfaces) (Teughels et al., 2006).

### 2.2 Ecological dysbiosis and pathogenic microbial communities

When oral hygiene maintenance is inadequate, leading to significant accumulation of plaque biofilm at the implant–abutment connection and restoration margins, the microenvironment undergoes marked alterations: oxygen partial pressure decreases, while gingival crevicular fluid (GCF) secretion increases, creating favorable conditions for the proliferation of anaerobic bacteria (Marsh and Zaura, 2017; Berglundh et al., 2018a). This triggers microbial dysbiosis, characterized by a shift in the flora structure from a symbiotic community dominated by Gram-positive bacteria to a pathogenic community predominantly composed of Gram-negative anaerobic bacteria (Sahrmann et al., 2020).

Similar to periodontitis in natural teeth, various core pathogenic bacteria have been confirmed to be closely associated with peri-implantitis. In addition to the classic “red complex”—Porphyromonas gingivalis, Tannerella forsythia, and Treponema denticola—high-throughput sequencing technologies have also revealed significant enrichment of bacteria such as Prevotella intermedia, Fusobacterium nucleatum, and Selenomonas spp. in peri-implantitis lesions (Banu Raza et al., 2022). Among these, *P. gingivalis* is regarded as a “keystone pathogen.” Even at relatively low abundance, it can secrete virulence factors such as proteases to disrupt the host’s primary immune defenses, thereby remodeling the entire microbial community and creating favorable conditions for the proliferation of other pathogenic bacteria (Hajishengallis et al., 2012; Chen et al., 2022b).

Furthermore, recent advancements have introduced the theory of the “competitive balancing effect” within the peri-implantitis-associated microbiota (Di Spirito et al., 2024a). This theory posits that the dysbiotic microbiome is not merely a chaotic overgrowth of bacteria, but rather a highly structured community where various pathogenic taxa compete for nutrients and ecological niches, ultimately reaching a dynamic, resilient equilibrium. This competitive balance allows the biofilm to maintain its collective pathogenicity and structural integrity even under environmental stress. These bacteria collaborate to form a biofilm with intricate structure and functional properties. The extracellular polymeric substance (EPS) matrix on the outer layer of the biofilm effectively impedes the recognition and elimination by host immune cells, while significantly reducing the penetration efficiency of antibiotics and other antimicrobial agents, thereby establishing an effective physicochemical barrier (Socransky and Haffajee, 2002). Critically, this EPS barrier facilitates long-term bacterial infection persistence by creating distinct oxygen and nutrient gradients. Bacteria deep within the biofilm structure transition into dormant, phenotypically resistant “persister” states that easily survive standard mechanical and chemical therapies (Niu et al., 2024). Furthermore, the complex micro-topography of the titanium surface acts as a sanctuary for these persistent biofilms, rendering complete eradication highly challenging and establishing the biological foundation for the chronic nature of the disease (Wang et al., 2025).

### 2.3 Pathological progression: from mucosal inflammation to peri-implantitis

Microbial imbalance constitutes the initial link in the onset of peri-implantitis, while the excessive activation and regulatory imbalance of the host immune system are the core mechanisms that directly lead to tissue damage. The pathological process begins when host cells recognize pathogen-associated molecular patterns (PAMPs), such as bacterial lipopolysaccharides (LPS) and peptidoglycan, through pattern recognition receptors (mainly Toll-like receptors TLR2 and TLR4), thereby activating key signaling pathways such as NF-κB. This prompts immune cells such as epithelial cells, fibroblasts, and macrophages to release a series of pro-inflammatory cytokines and chemokines, including interleukin-1β (IL-1β) and tumor necrosis factor-α (TNF-α) (Cochran, 2008; Berezow and Darveau, 2011; Meyle and Chapple, 2015). These inflammatory mediators trigger local vascular reactions and the recruitment of inflammatory cells such as neutrophils. The clinical manifestations are redness and swelling of the peri-implant mucosa and bleeding on probing, which is the stage of peri-implant mucositis. At this stage, the inflammation is still reversible. Timely intervention can restore tissue homeostasis.

If the inflammation continues to progress, the immune response will gradually shift to a destructive mode. While neutrophils are phagocytosing and eliminating pathogens, they release proteolytic enzymes such as matrix metalloproteinases (MMPs), leading to the degradation of the extracellular matrix and the destruction of soft tissue barriers (Sorsa et al., 2016). With the activation of adaptive immunity, cell subsets such as Th1 and Th17 differentiate in the inflammatory microenvironment and produce a large number of cytokines such as interleukin-17, further intensifying the inflammatory response (Carcuac and Berglundh, 2014). At the cellular and molecular levels, inflammatory factors stimulate osteoblasts, fibroblasts, and activated T cells to highly express receptor activator of nuclear factor kappa-B ligand (RANKL), while inhibiting the synthesis of its natural antagonist osteoprotegerin (OPG), leading to an imbalance in the RANKL/OPG system (Liu et al., 2019). The disorder of this signaling pathway strongly drives the differentiation and activation of osteoclasts, disrupts the metabolic balance of bone tissue, and ultimately leads to progressive bone loss, marking the entry of the disease into the irreversible stage of peri-implantitis. Crucially, alongside immune hyperactivation, the dysbiotic biofilm actively orchestrates an immune suppression network to ensure its survival. Key peri-implant pathogens subvert local host defenses by secreting proteases that degrade complement proteins and immunoglobulins (Hajishengallis et al., 2013). Furthermore, these pathogens manipulate local immune cell responses, driving macrophage polarization toward a tolerant, non-bactericidal state and stimulating the recruitment of immunosuppressive cell populations, such as myeloid-derived suppressor cells (MDSCs) and regulatory T cells (Tregs) (Cafferata et al., 2025; Jang et al., 2026). This localized immune subversion creates a paradoxical microenvironment: the host immune system is paralyzed in its ability to effectively clear the infection, yet remains sufficiently activated to continuously release bone-resorbing cytokines. This persistent infection-inflammation loop is a core mechanism driving the relentless progression of peri-implantitis (Jang et al., 2026).

As highlighted by recent high-quality reviews in the broader field of implant infections, the presence of a biomaterial dramatically alters the host–pathogen dynamic, leading to profound and aggressive immune dysregulation (Masters et al., 2022). Although extensively studied in orthopedic periprosthetic joint infections, these core mechanisms are highly translatable to the peri-implant microenvironment in the oral cavity. Pathogens colonizing the implant surface construct robust biofilms that not only restrict antimicrobial diffusion but also physically impede immune cell penetration. Crucially, these pathogenic communities actively orchestrate a localized immune suppression network; they subvert host defenses by skewing macrophage polarization toward a tolerant phenotype and stimulating the recruitment of immunosuppressive populations, such as MDSCs. Furthermore, bacteria can persist intracellularly or invade the osteocyte lacuno-canalicular network (OLCN) of the surrounding bone. This unique combination of immune paralysis and deep osseous invasion explains the accelerated, persistent trajectory of bone loss (osteolysis) observed around implants, underscoring why traditional mechanical debridement is often insufficient and highlighting the urgent need for targeted immunomodulatory interventions (Masters et al., 2022).

**Figure 1:**
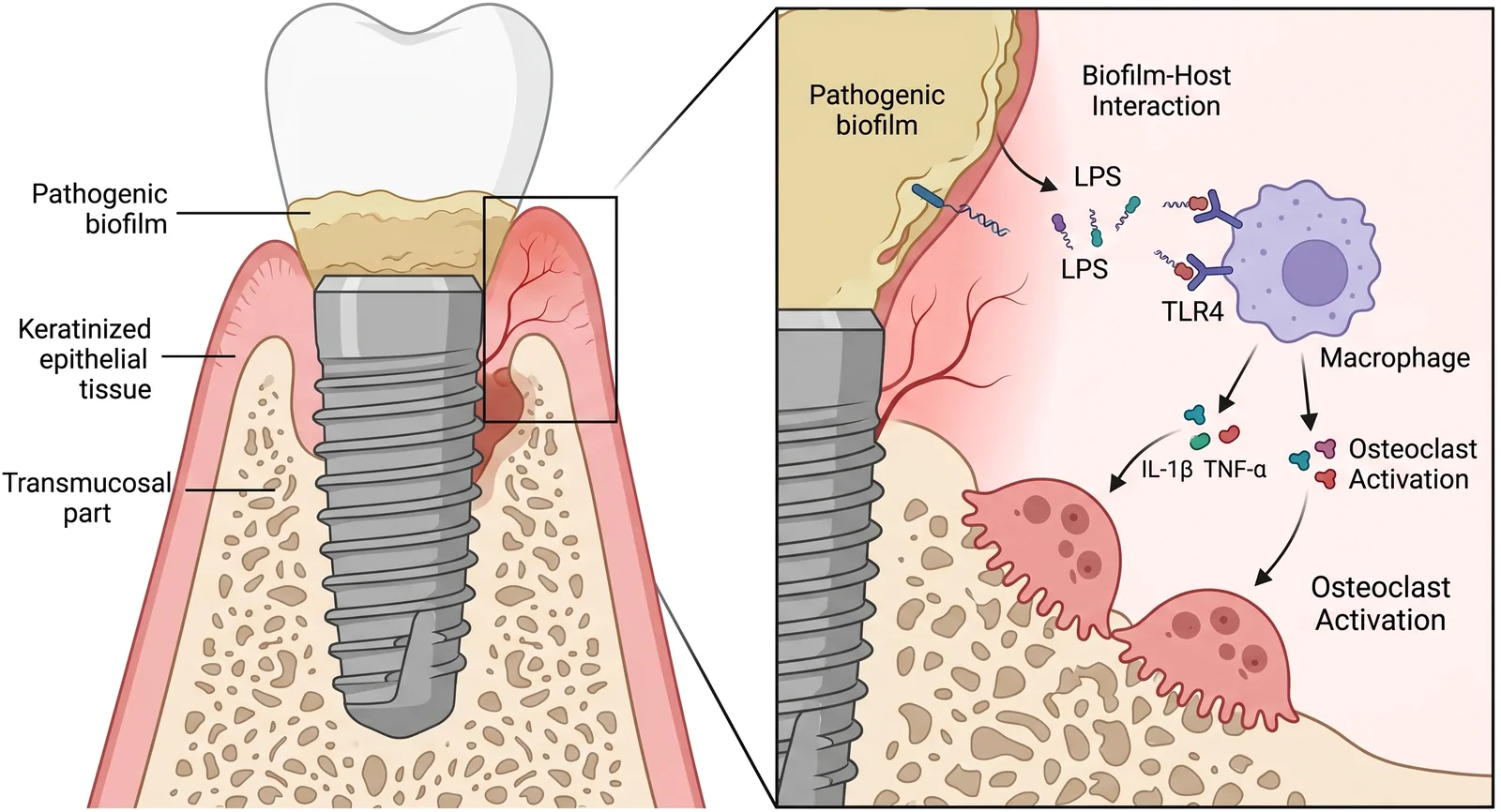
Schematic illustration of the core immunopathological mechanisms in peri-implantitis. The diagram depicts a dental implant with an active bone defect covered by a pathogenic biofilm. At the cellular level, the biofilm-host interaction is primarily driven by bacterial lipopolysaccharides (LPS) binding to Toll-like receptor 4 (TLR4) on the surface of macrophages. This interaction initiates an intracellular signaling cascade that triggers the release of pro-inflammatory cytokines, specifically interleukin-1 beta (IL-1β) and tumor necrosis factor-alpha (TNF-α). These cytokines subsequently upregulate the RANKL/OPG pathway, activating osteoclasts and leading to progressive alveolar bone resorption.

In summary, keystone pathogens such as *P. gingivalis* activate the host’s TLR4 receptors via virulence factors, initiating the NF-κB signaling pathway and leading to the overproduction of pro-inflammatory cytokines, notably IL-1β and TNF-α. This localized release of inflammatory cytokines serves as a primary driver of the subsequent tissue-destructive cascade (**Figure 1**). This process elucidates the continuous pathological chain from microbial challenge to dysregulated host immune response, culminating in peri-implantitis.

## 3 Research progress in early diagnosis methods for peri-implantitis

The goal of early diagnosis is to identify high-risk sites and biologically active lesions before irreversible bone tissue destruction occurs or progresses significantly. The development of diagnostic techniques is undergoing a profound transformation from macroscopic clinical manifestations to microscopic molecular early warning.

### 3.1 Refinement of routine clinical diagnosis

Traditional clinical examination remains the cornerstone for diagnosing peri-implantitis to this day, and its application is moving towards refinement and standardization. In terms of clinical indicators, BOP serves as the core parameter for evaluating the active inflammatory state of soft tissues, while PPD is a critical measure for assessing attachment loss and structural tissue destruction. Their measurement results are influenced by multiple factors such as probing pressure, angle, probe type, and prosthesis morphology (Lang et al., 1986). To enhance the consistency and comparability of diagnosis, it is currently recommended to use standardized periodontal probes with pressure control function (usually set at 0.25 N) for measurement (Barendregt et al., 1996). According to the 2017 World Workshop consensus, a definitive diagnosis of peri-implantitis requires the presence of bleeding and/or suppuration on probing, an increased probing depth compared to previous examinations, and progressive bone loss beyond the initial physiological remodeling (Berglundh et al., 2018a). In the absence of baseline clinical and radiographic data, the diagnosis is confirmed by the presence of BOP, probing depths of ≥6 mm, and marginal bone levels ≥3 mm apical to the most coronal portion of the intraosseous part of the implant, typically evaluated after the first year of functional loading (Berglundh et al., 2018a).

In terms of imaging technology, periapical radiographs represent the first-line standard for routine monitoring of marginal bone levels (Sahrmann et al., 2024). Panoramic tomography may be used as an adjunctive screening tool but lacks the site-specific resolution of periapical films and is not recommended as the primary modality for assessing peri-implant bone levels (Sewerin and Skov, 1992). Their limitations lie in the potential for image overlap to obscure buccolingual bone resorption, and they typically require approximately 30% loss of bone mineral density for clear detection. Cone beam computed tomography (CBCT) holds significant value in diagnosis and surgical planning due to its ability to provide three-dimensional imaging, enabling accurate assessment of the morphology, extent, and volume of bone defects (Haas et al., 2017; Jacobs et al., 2024). However, to avoid over-interpretation and unnecessary radiation exposure, CBCT should not be used as a routine diagnostic tool for peri-implantitis (Bornstein et al., 2014). It is specifically indicated only when two-dimensional radiographs are inconclusive or when a precise three-dimensional evaluation of the bone defect is required prior to complex surgical or regenerative interventions (Mandelaris et al., 2017). While this technology poses challenges such as radiation exposure and metal artifacts, its clinical application is gradually being optimized through the development of low-dose scanning protocols and improvements in artifact correction algorithms. Furthermore, artificial intelligence (AI) technology has begun to be applied in the field of imaging analysis. Deep learning-based algorithms can automatically identify and measure marginal bone levels in CBCT or periapical radiographs, and can even detect early-stage bone changes by analyzing trabecular texture features. Relevant studies have shown that their diagnostic accuracy is comparable to that of professional radiologists (Abbott et al., 2024; Luke and Rezallah, 2025).

### 3.2 Molecular diagnostic techniques

Molecular diagnostics focuses on detecting pathogenic microorganisms or the host response markers they induce, aiming to achieve early and highly sensitive disease warning. In the field of microbial detection, real-time quantitative polymerase chain reaction (qPCR) technology enables rapid quantitative analysis of specific core pathogenic bacteria (such as *P. gingivalis*) in GCF samples. This method offers advantages such as fast detection speed and controllable costs, making it suitable for dynamic monitoring of specific pathogen loads in clinical settings (Persson and Renvert, 2013). Meanwhile, high-throughput sequencing technology based on the 16S ribosomal RNA (rRNA) gene provides a comprehensive structural map of the peri-implant microbial community, enabling not only the identification of bacterial species composition but also a systematic assessment of microbial diversity, structural characteristics, and relative abundance (Schloss et al., 2009). By constructing microbial dysbiosis indices or identifying specific pathogenic microbial networks, this technology enables comprehensive assessment of disease risk and status, with its clinical translation potential becoming increasingly prominent as technical costs decline.

In terms of host-derived biomarker detection, peri-implant crevicular fluid (PICF) and saliva serve as liquid biopsy samples reflecting host–microbial interactions, containing a wealth of biological information molecules. Among these, pro-inflammatory cytokines such as IL-1β and TNF-α serve as direct indicators of inflammatory activity. Their concentrations in patients with peri-implantitis are significantly higher than those in healthy controls and individuals with peri-implant mucositis, providing a quantitative basis for assessing inflammatory status (Duarte et al., 2016; Kalsi et al., 2021).

Matrix metalloproteinase-8 (MMP-8), particularly its active form (aMMP-8), has demonstrated significant diagnostic value through multiple studies [47]. While initial research highlights aMMP-8 as a highly promising biomarker strongly correlated with the rate of connective tissue destruction, critical analysis reveals ongoing controversies regarding its universal clinical application (Räisänen et al., 2023). Although point-of-care tests utilizing aMMP-8 have shown favorable diagnostic performance (Fragkioudakis et al., 2024), establishing a definitive clinical threshold remains complicated by variations in sampling techniques, local fluid volumes, and patient-specific baseline inflammatory states. Furthermore, while the sensitivity of aMMP-8 for detecting active tissue breakdown is generally reported to be high, its specificity can be significantly compromised by the presence of gingivitis, periodontitis, or other concurrent oral inflammatory conditions. A meta-analysis reported that the specificity of aMMP-8 POCT is approximately 0.64, indicating a substantial false-positive rate when used as a standalone diagnostic tool (Zhang et al., 2025). Importantly, aMMP-8 POCT has been shown to be unable to reliably differentiate between gingivitis and periodontal health, and its diagnostic accuracy is further reduced in patients with systemic inflammatory conditions such as Crohn’s disease (Rautava et al., 2020; Deng et al., 2021). Smoking habits and variations in periodontal status also influence test performance (Yilmaz et al., 2023). This lack of absolute specificity necessitates careful clinical correlation rather than relying on isolated threshold values.

Similarly, the ratio of RANKL to OPG directly reflects the state of bone metabolic balance. An increase in this ratio indicates enhanced or ongoing bone resorption activity, providing a crucial early warning signal for clinical assessment of bone metabolic disorders (Theodoridis et al., 2021; Ateeq et al., 2022). However, the clinical utility of the RANKL/OPG ratio is currently limited by significant inter-individual variability and the lack of standardized diagnostic cut-off values. A systematic review and meta-analysis emphasized this limitation, revealing that while RANKL levels tended to be higher in peri-implantitis, the difference from healthy sites did not reach statistical significance (*P* = 0.078), and no significant differences were found for OPG and the RANKL/OPG ratio (Theodoridis et al., 2021). Consequently, there is only limited evidence that these biomarkers can serve as strong standalone indicators for distinguishing between healthy and diseased peri-implant tissues, and the statistical overlap in concentration ranges between peri-implant health, mucositis, and peri-implantitis remains a major research controversy (Theodoridis et al., 2021). Therefore, while these molecular biomarkers provide critical early warning signals, their true diagnostic accuracy and clinical translation rely heavily on their integration with conventional clinical parameters rather than standalone diagnostic thresholds.

### 3.3 New diagnostic trends: instant and intelligent

The development of POCT represents a critical direction for translating molecular diagnostics into clinical practice. It aims to streamline complex laboratory procedures into workflows that can be completed within 15–30 minutes in a clinical setting. Currently, a rapid test kit based on immunochromatography for detecting aMMP-8 has been commercialized and demonstrated favorable diagnostic performance in clinical evaluations (Lähteenmäki et al., 2022). Concurrently, multi-parameter joint detection platforms based on microfluidic chip technology, electrochemical sensing, and optical detection principles are also in active research and development (Dhall et al., 2022; Bhardwaj et al., 2024; Ghahramani et al., 2025). The future trend of diagnostic technology will move beyond the detection of single biomarkers and shift toward the systematic integration of multi-omics data. By combining clinical parameters, microbiome, proteome, metabolome, and even genomic data, and utilizing machine learning algorithms to construct high-precision disease prediction models and individualized risk scoring systems, it will not only enable early disease diagnosis but also facilitate the assessment of disease risk, prediction of progression trajectories, and evaluation of treatment responses. This advancement will thereby drive the transformation of diagnostic and therapeutic models toward predictive, preventive, and personalized precision medicine (Sun et al., 2022).

**Figure 2:**
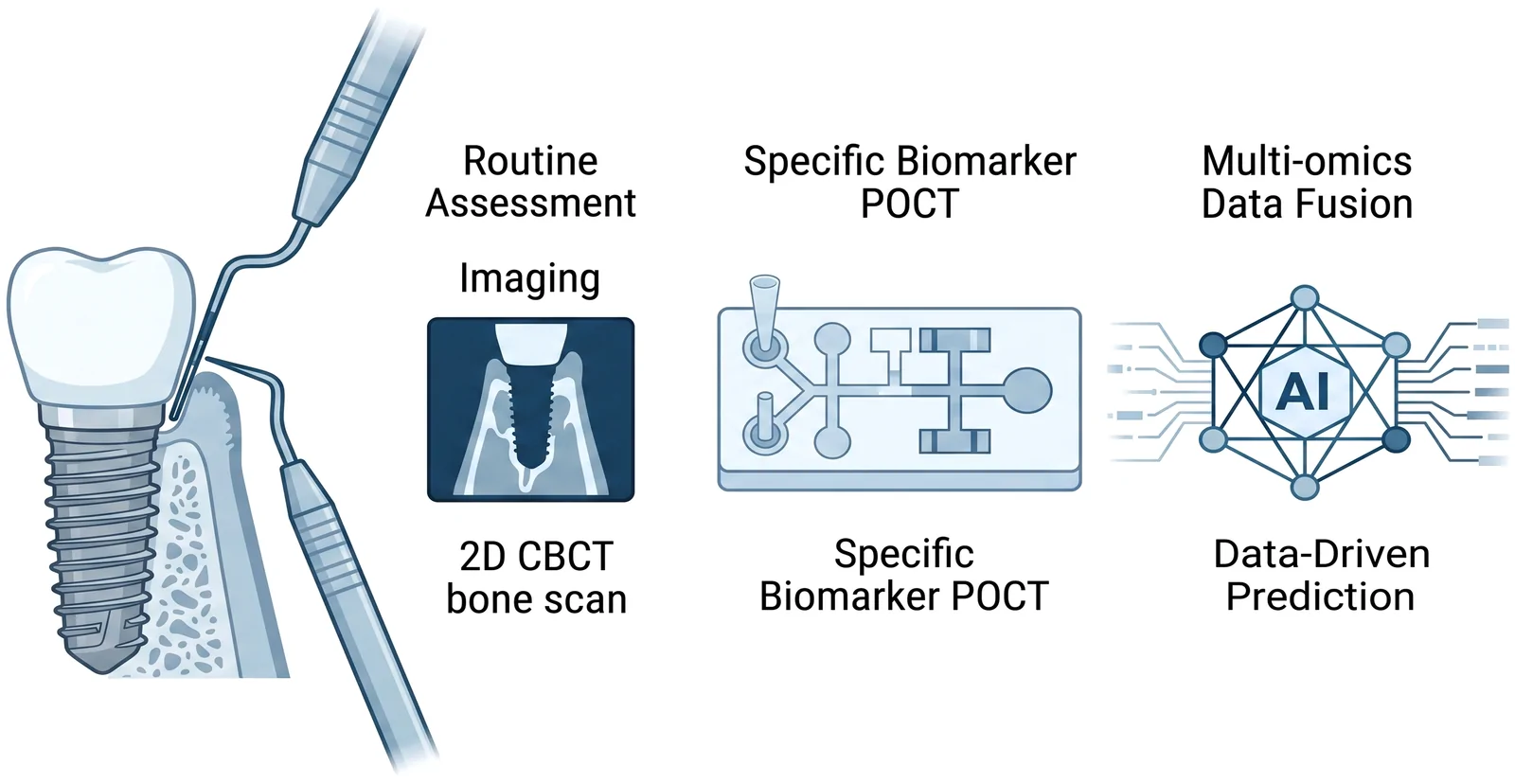
The evolving landscape of diagnostic technologies for peri-implantitis. This schematic illustrates the paradigm shift in diagnostic strategies, progressing from traditional clinical assessments (e.g., periodontal probing and 2D/3D radiographic bone scans) toward highly specific molecular diagnostics. The figure highlights the integration of biomarker point-of-care testing (POCT) utilizing advanced microfluidic chips. Furthermore, the future trajectory emphasizes data-driven prediction models powered by artificial intelligence (AI) and multi-omics data fusion.

In conclusion, the early diagnostic techniques for peri-implantitis are undergoing a paradigm shift from macroscopic to microscopic and from single to integrated. Its evolution path can be summarized as follows: routine diagnosis centered on clinical and imaging examinations, molecular diagnosis centered on the detection of microbial and host markers, and the future of intelligent diagnosis driven by multi-omics data fusion and AI. This development trend aims to achieve earlier, more accurate, and more convenient disease early warning and assessment (**Figure 2**).

## 4 Treatment strategies for peri-implantitis: from infection control to microecological regulation

With the deepening understanding of its etiology, the treatment philosophy for peri-implantitis has evolved from a traditional infection control model primarily focused on mechanical debridement to a new paradigm aimed at restoring the peri-implant microecological balance. Consequently, corresponding therapeutic strategies are also undergoing continuous updates and refinements.

### 4.1 Innovation in basic anti-infection treatment

The core objective of all therapeutic interventions lies in the effective removal of biofilm contamination and the achievement of biological decontamination on the implant surface, thereby establishing a favorable microenvironment for tissue healing.

In the field of non-surgical debridement, traditional metal scaling instruments, which carry the risk of scratching the titanium surface of implants and potentially increasing the reattachment of plaque biofilm, have gradually been replaced by instruments made of materials such as carbon fiber, titanium alloy, polymers, or polyetheretherketone (PEEK). Ultrasonic or piezoelectric ceramic systems, when used with non-metallic tips, can enhance the efficiency of biofilm removal. However, their application requires precise control of operating parameters and adequate cooling measures to avoid thermal damage to bone tissue. Air polishing technology, which utilizes low-abrasive powders (such as glycine or erythritol) and specialized nozzles, has been demonstrated to effectively and minimally invasively remove biofilm from implant and restoration surfaces while causing minimal damage to surrounding soft and hard tissues. Consequently, it has become one of the preferred techniques for both non-surgical and surgical debridement (Ramanauskaite et al., 2021).

For cases involving deep periodontal pockets or complex bone defects, flap surgery is often required to perform thorough debridement under direct vision. Methods for implant surface decontamination primarily include mechanical treatment (such as using titanium brushes), chemical treatment (such as applying citric acid or tetracycline solutions), and physical techniques. Among these, laser-assisted debridement (particularly Er: YAG laser) has attracted clinical attention due to its significant bactericidal effects and minimal damage to the implant surface structure (Wang et al., 2020; Chen et al., 2022a). However, a critical perspective must be maintained regarding these techniques. Despite the array of available instruments, achieving 100% biological decontamination on micro-rough implant surfaces and within complex thread geometries remains clinically unachievable (Ozgu and Ustun, 2025). This fundamental limitation highlights a major contradiction between highly controlled *in vitro* efficacy studies and the persistently high recurrence rates observed in real-world clinical practice, underscoring the reality that current anti-infective therapies primarily manage, rather than cure, the dysbiotic state.

Among auxiliary treatment modalities, photodynamic therapy (PDT) employs a synergistic interaction between a photosensitizer and light of a specific wavelength to generate bactericidal singlet oxygen, serving as a non-antibiotic antimicrobial strategy in clinical practice (Rkein and Ozog, 2014). For specific indications, local drug delivery systems, such as minocycline-loaded microspheres, can be utilized as adjuncts to non-surgical therapy to enhance localized drug concentration (Yoon et al., 2020). The use of systemic antibiotics should be strictly indicated and is generally recommended only for patients presenting with systemic symptoms of acute infection or those with compromised immune function. Ideally, their administration should be guided by microbial culture and drug susceptibility testing.

### 4.2 Emerging strategies for microecological regulation

The ultimate goal of anti-infective therapy is not to achieve a sterile state, but to reconstruct a healthy microecology that coexists with the host. Probiotic therapy, administered orally or topically with specific strains (such as *Lactobacillus reuteri*), promotes the restoration of microbial balance through mechanisms including competitive colonization, secretion of antimicrobial metabolites (e.g., *reuterin*), and modulation of host immune responses. Clinical studies have confirmed the positive role of this therapy in the prevention and adjunctive treatment of peri-implant mucositis (Flichy-Fernández et al., 2015). Furthermore, quorum sensing inhibitors (QSIs) disrupt the bacterial communication system that relies on signaling molecules to coordinate virulence expression and biofilm formation, thereby attenuating their collaborative pathogenicity. These inhibitors have demonstrated promising anti-biofilm potential in preclinical studies (Brackman and Coenye, 2015). Meanwhile, as a class of endogenous peptide molecules, targeted antimicrobial peptides (AMPs) offer a novel strategic direction for eliminating pathogenic microorganisms while preserving the stability of commensal flora, owing to their broad-spectrum bactericidal activity and low propensity for inducing resistance.

Beyond direct antimicrobial strategies, advanced immunomodulatory therapies are currently being developed to actively reverse the localized immune suppression network. Recognizing the detrimental accumulation of immunosuppressive cell populations—specifically MDSCs and the dysregulation of Tregs in peri-implantitis lesions—novel biomaterials are being designed to reprogram the local host response (Cafferata et al., 2025; Jang et al., 2026). These immunomodulatory scaffolds aim to deplete or functionally alter MDSCs and restore the delicate Treg/Th17 balance, thereby re-establishing a competent yet controlled immune microenvironment that favors tissue healing over chronic inflammation and bone resorption (Zhang et al., 2024).

### 4.3 Stepped and personalized treatment concepts

In clinical practice, it is recommended to adhere to the “Cumulative Interceptive Supportive Therapy” or a stepped treatment approach. The selection of a treatment plan should be based on a comprehensive assessment of disease severity, bone defect morphology, and the patient’s systemic and local risk factors.

For the early stage characterized solely by peri-implant mucositis, the core of treatment lies in etiological control. This primarily involves enhanced oral hygiene instruction, implementation of professional plaque removal measures, and the elimination of all potential plaque-retentive factors, such as defective restoration margins or morphology. When the condition progresses to early peri-implantitis, non-surgical intervention should be prioritized. Modified debridement techniques, including ultrasonic scaling, air abrasion, and laser-assisted therapy, can be employed for management (Suárez-López Del Amo et al., 2016).

When the disease progresses to the moderate-to-severe peri-implantitis stage, as evidenced by persistent probing depths of ≥6 mm with BOP following non-surgical intervention, surgical treatment becomes necessary (Berglundh et al., 2018a). Surgical options include flap debridement, resectional surgery (osseous recontouring/implantoplasty), or regenerative surgery. The choice of procedure depends on the morphology of the bone defect and the patient’s needs (Renvert et al., 2018). Throughout the treatment process, it is essential to manage the patient’s individual factors, such as smoking status and glycemic control in diabetes, as these are important determinants of treatment efficacy and disease recurrence (Monje et al., 2015).

### 4.4 Microbiological shifts following peri-implant therapy and clinical implications

A critical aspect of understanding the efficacy of peri-implantitis treatments lies in monitoring the dynamic changes in the peri-implant microbiome following intervention. Recent comprehensive reviews have demonstrated that both non-surgical and surgical therapies induce significant, albeit often temporary, alterations in the microbial composition of the peri-implant pocket (Di Spirito et al., 2024a). Following mechanical debridement and surface decontamination, there is typically a marked reduction in the total bacterial load and a significant decrease in the relative abundance of classical red-complex pathogens and Gram-negative anaerobes (Di Palo, 2025).

Crucially, these microbiological variations directly translate into changes in peri-implant clinical, radiographic, and crevicular parameters. A successful shift toward a more symbiotic microbiome—characterized by the repopulation of health-associated Gram-positive cocci—correlates strongly with the resolution of BOP, a reduction in PPD, and a decrease in pro-inflammatory biomarkers (such as aMMP-8 and IL-1β) within the PICF (Banu Raza et al., 2022; Anuntakarun et al., 2025). Conversely, a failure to significantly alter the microbial profile, or a rapid rebound of the dysbiotic community, is highly predictive of persistent soft tissue inflammation and continued radiographic marginal bone loss (Di Spirito et al., 2024b).

In the context of post-treatment microbial dynamics, the newly identified potential role of *Fusobacterium nucleatum* has garnered significant attention. While interventions may effectively eradicate specific keystone pathogens, *F. nucleatum* often demonstrates remarkable resilience and persistence post-therapy (Di Spirito et al., 2024a). Acting as a critical “bridge” species, its survival allows it to co-aggregate with both early colonizers and late pathogenic colonizers (Pignatelli et al., 2023). Consequently, the persistence of *F. nucleatum* after treatment serves as a scaffolding mechanism that accelerates the recolonization of other anaerobic pathogens, thereby playing a pivotal role in treatment resistance and the high recurrence rates associated with peri-implantitis (Di Spirito et al., 2024a). Therefore, monitoring and specifically targeting the resilient survival of *F. nucleatum* post-therapy may represent a critical new frontier in achieving long-term microecological stability.

### 4.5 Comparative efficacy of treatment modalities: evidence from clinical and preclinical studies

To bridge the gap between theoretical mechanisms and clinical application, it is imperative to comprehensively evaluate the efficacy of current interventions. Recent evidence from both randomized controlled trials (RCTs) and *in vivo* animal models highlights the varied success rates and limitations of different therapeutic modalities (**Table 1**). In non-surgical and adjunctive therapies, RCTs have demonstrated that while probiotics such as *Lactobacillus reuteri* can modulate the microecology and provide benefits for peri-implant mucositis, mechanical debridement combined with laser applications (e.g., Er: YAG lasers) yields significant, albeit sometimes short-term, microbiological changes and clinical improvements in established peri-implantitis lesions (López-Valverde et al., 2024; Al-Zawawi, 2025; Bengtsson et al., 2025). Preclinical models remain crucial for testing novel approaches before clinical translation; for instance, studies in beagle dogs have validated the localized efficacy of sustained-release minocycline agents in reducing pocket depth and inflammation (Yoon et al., 2020).

**Table 1:** Summary of pivotal clinical and preclinical studies evaluating peri-implantitis treatment modalities.

Study type	Subject/model	Intervention evaluated	Key findings and clinical implications	References
RCT	Human, peri-implant mucositis (N=34 patients, 77 implants)	Probiotic (*L. reuteri*)	Significant reduction in BOP, GI, and PPD at 30 days. Probiotic therapy acts as an effective adjunct for controlling peri-implant mucosal inflammation.	(MeyleChapple, 2015)
RCT	Human, peri-implantitis (N=23)	Er : YAG laser debridement	At 6 months: PD reduction 0.84 mm (laser) vs. 0.41 mm (control); buccal site: 1.31 mm vs. 0.25 mm (p=0.037). Anaerobic bacteria reduced only in control group. Laser offers additive benefit for PD reduction at specific sites.	(López-Valverde et al., 2024)
RCT	Human, peri-implantitis (N=24)	Laser-assisted regenerative surgery + GBR	At 6 months: PD reduction 2.65 ± 2.14 mm (laser) vs. 1.85 ± 1.71 mm (control), p=0.014; bone gain 1.27 ± 1.14 mm vs. 1.08 ± 1.04 mm (p=0.666). Major membrane exposure impaired CAL gain (1.03 vs. 2.47 mm, p=0.051).	(Luke and Rezallah, 2025)
Multicenter RCT	Human, peri-implantitis (N=58, 63 implants)	Reconstructive therapy (Electrolysis vs. Hydrogen peroxide)	Both methods achieved comparable 12-month clinical improvements (MBL gain >1.6 mm); GS showed a trend toward higher disease resolution (87.5% vs. 64.5%, OR=3.76, p=0.088). Wider keratinized mucosa significantly predicted higher resolution (OR=2.21, p=0.009).	(Renvert and Polyzois, 2015)
Preclinical	Beagle dog (N=6 dogs, 24 implants)	Local minocycline: CA microspheres (MC) vs. PG microspheres	At 8 weeks: PPD reduction 3.81→2.00 mm (MC), 3.44→2.42 mm (PG). CA carriers showed prolonged retention (p<0.017); MC showed longer bacteriostatic effect (9.33 vs. 1.17 days, p<0.05).	(Masters et al., 2022)
Preclinical	Rat model (N=15 rats)	UCB-Exos loaded in nGA hydrogel	UCB-Exos significantly increased BV/TV, BMD, Tb.N, Tb.Th; downregulated TNF-α and IL-1β, upregulated IL-10. Mechanistically, delivered miR-182-5p directly targeted MYD88 3’UTR to inhibit NF-κB signaling, promoting osteogenesis.	(Rkein and Ozog, 2014)

In the realm of surgical and regenerative treatments, recent multicenter RCTs comparing surface decontamination protocols (e.g., electrolysis vs. hydrogen peroxide) demonstrated comparable bone level gains (>1.6 mm) at 12 months. Crucially, these studies underscore that the width of the keratinized mucosa acts as a significant continuous predictor for definitive disease resolution (odds ratio [OR] = 2.21), highlighting the necessity of comprehensive soft tissue management alongside surface decontamination (Monje et al., 2025). Meanwhile, cutting-edge animal studies are laying the groundwork for future regenerative strategies. For example, rat models of peri-implantitis have recently been utilized to demonstrate that umbilical cord blood-derived exosomes can successfully deliver microRNAs (e.g., miR-182-5p) to therapeutically target the NF-κB inflammatory pathway, offering a cell-free alternative to traditional bone grafting (Liu et al., 2025). By comprehensively comparing these clinical and preclinical findings, it becomes evident that future research must prioritize combinatorial approaches—integrating advanced surface decontamination with targeted, smart biomaterials—to achieve long-term functional stability.

## 5 Frontier advances in the regeneration treatment of peri-implantitis

The transition from infection control to regenerative therapy is not merely sequential but deeply interconnected. As established in Section 2, the pathological cascade of peri-implantitis is driven by a dysbiotic microbial biofilm that perpetuates a destructive host inflammatory response. Therefore, attempts at tissue regeneration are frequently compromised unless the pathogenic microenvironment is first effectively managed. The principles of regenerative therapy are fundamentally based on creating a biological milieu conducive to osteogenesis—a state that is incompatible with the pro-inflammatory, tissue-degrading conditions induced by residual pathogens. The primary objective of regenerative therapy is thus two-fold: first, to achieve definitive biological decontamination of the implant surface and restore microecological homeostasis; second, on this foundation, to guide predictable new bone formation.

For peri-implantitis with moderate to severe bone defects, infection control alone is insufficient to restore its biomechanical support function and long-term stability. The primary objective of regenerative therapy is to eliminate the inflammatory environment through thorough debridement and, on this basis, guide new bone formation to achieve functional integration between the implant and surrounding bone tissue, thereby restoring its physiological support and load-bearing capacity. Crucially, the success of this functional reconstruction is fundamentally predicated on the restoration of microecological homeostasis. The regenerative microenvironment is continuously challenged by the preceding microbial dysbiosis; thus, transitioning from a pathogenic biofilm to a symbiotic microflora is not merely an endpoint of infection control, but an absolute prerequisite for tissue engineering techniques to succeed. Without establishing a microbiologically stable niche, newly regenerated tissues remain highly susceptible to rapid reinfection and structural degradation.

### 5.1 Core technology and materials: guided bone regeneration

**Figure 3:**
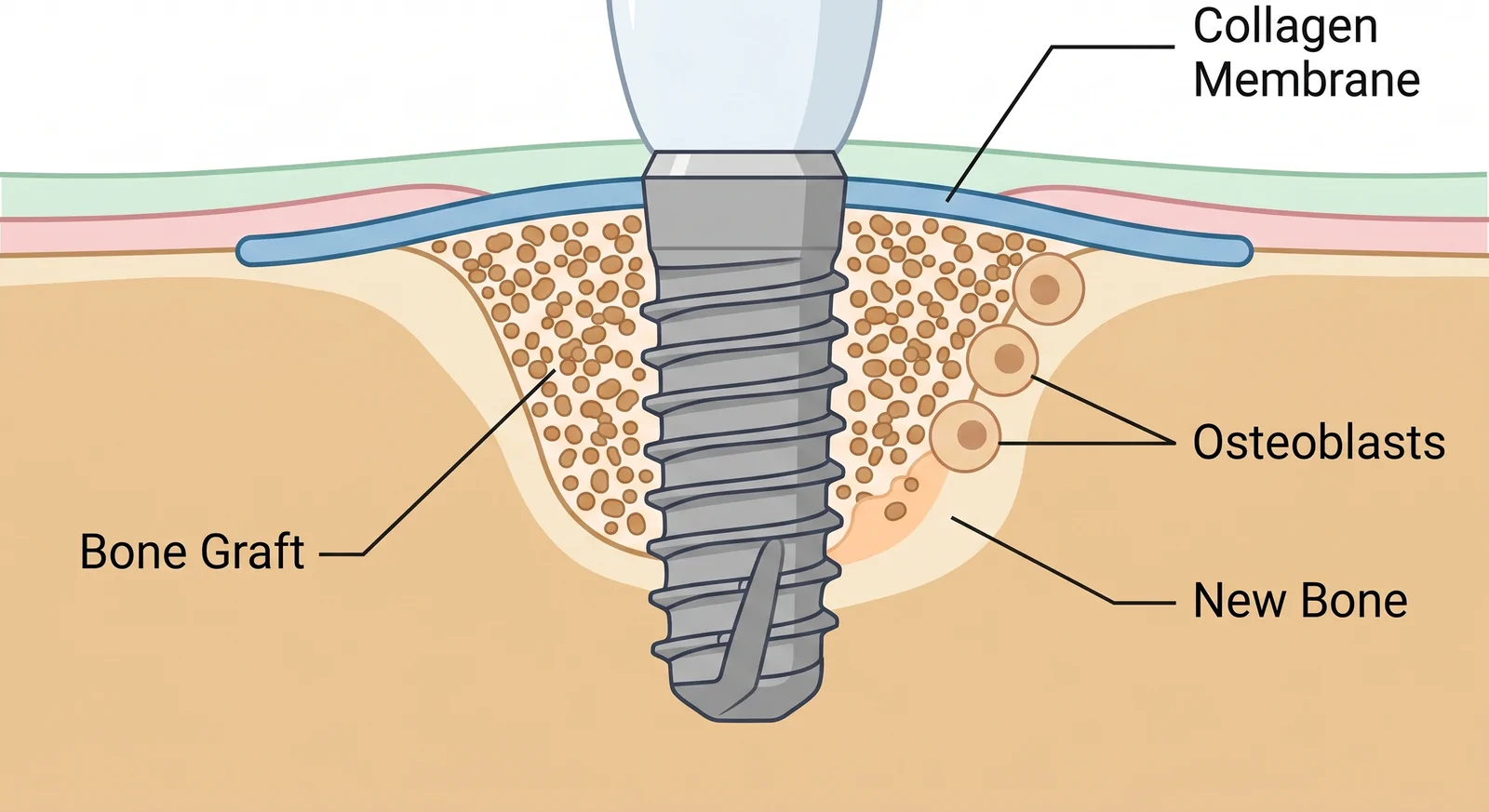
Schematic diagram of guided bone regeneration (GBR) for peri-implant bone defects. This illustration provides a cross-sectional view of an implant situated within an alveolar bone defect managed via the GBR protocol. The defect is precisely filled with osteoconductive bone graft particles and securely isolated by a resorbable collagen barrier membrane, which prevents epithelial downgrowth. Mononuclear osteoblasts are depicted actively depositing new bone matrix at the defect margins, facilitating predictable functional reconstruction and structural osseointegration.

GBR is currently a core technique for the regenerative treatment of peri-implant bone defects. Its fundamental principle involves the use of a barrier membrane to create an isolated and stable space within the bone defect area, preventing the preferential infiltration of epithelial cells and connective tissue. This provides essential conditions for osteogenic cells to proliferate and differentiate within the region, ultimately achieving directional regeneration of bone tissue (Elgali et al., 2017) (**Figure 3**).

In clinical practice, bone grafting materials are often used in combination with barrier membranes. Bone grafting materials primarily serve as scaffolds for new bone ingrowth, including autogenous bone, allograft, xenograft (such as deproteinized bovine bone mineral [DBBM]), and synthetic bone materials (e.g., β-tricalcium phosphate and hydroxyapatite composites). Among these, autogenous bone exhibits osteogenic, osteoinductive, and osteoconductive properties, although its harvesting increases surgical trauma and time. Xenogeneic substitutes like DBBM mainly provide a stable three-dimensional structure through osteoconduction. Synthetic materials have garnered attention due to their controllable degradation rates and excellent biocompatibility.

Barrier membranes are categorized into non-resorbable and resorbable types. Non-resorbable membranes, such as dense polytetrafluoroethylene (d-PTFE) membranes, offer strong space-maintaining capabilities but require a secondary surgical procedure for removal (Vroom et al., 2022). Resorbable membranes, predominantly collagen-based, are widely used due to their favorable biocompatibility and elimination of the need for secondary surgery (Abtahi et al., 2023). Current research focuses on extending the barrier function duration of resorbable membranes through physical or chemical cross-linking techniques, as well as developing functionalized membrane materials with antimicrobial properties or growth factor release capabilities to enhance their biological activity. By incorporating targeted antimicrobial agents or probiotic metabolites into the membrane matrix, these smart biomaterials can actively modulate the local microecology during the critical early healing phase, preventing the recolonization of Gram−negative anaerobes and protecting the underlying osteogenic process from bacteria−induced inflammatory resorption (Li et al., 2025).

### 5.2 Bioactive adjuncts: accelerating and optimizing regeneration

To enhance the predictability of GBR outcomes, various bioactive adjuncts have been introduced into clinical research and practice. The core objective of these adjuncts is to provide an optimized microenvironment for cell recruitment, proliferation, and differentiation within the bone defect. Among them, recombinant human platelet-derived growth factor-BB (rhPDGF-BB) and recombinant human bone morphogenetic protein-2 (rhBMP-2) have been most extensively studied and have received approval from the U.S. Food and Drug Administration (FDA) (Aghaloo et al., 2025). rhPDGF-BB promotes angiogenesis and cellular chemotaxis, while rhBMP-2 exhibits significant osteoinductive activity (Galarraga-Vinueza et al., 2023). Their combined use with bone grafting materials has been confirmed in some clinical studies to enhance bone regeneration. However, the high cost and required supraphysiological dosages limit their widespread clinical application.

Platelet-rich fibrin (PRF), as a second-generation autologous platelet concentrate, is characterized by simple preparation and high safety. It contains abundant growth factors and leukocytes, and forms a three-dimensional fibrin network. When mixed with bone grafting materials or used as a covering membrane, it is believed to promote early healing and maturation of both soft and hard tissues (Miron et al., 2017).

Moving beyond conventional GBR, the field is rapidly pivoting toward cutting-edge tissue engineering and intelligent biomaterials. 3D-printed smart scaffolds represent a major breakthrough, allowing for the fabrication of patient-specific constructs with hierarchical porosity that perfectly match complex multi-wall bone defects (Ivanovski et al., 2024; Lee et al., 2025). Crucially, these advanced scaffolds are evolving from passive space-maintainers into dynamic, stimuli-responsive platforms (Bai et al., 2025). Researchers are actively developing sound-, light-, and magnetic field-responsive antimicrobial materials. For instance, piezoelectric or magnetoelectric nanoparticles embedded within hydrogels can generate localized reactive oxygen species (ROS) or release antimicrobial peptides strictly on-demand when subjected to external ultrasound or magnetic fields (Yang et al., 2024). Similarly, near-infrared (NIR) light-responsive systems allow for non-invasive, targeted eradication of persistent biofilm residing deep within the defect site during the critical early stages of healing, ensuring a sterile osteogenic niche without the need for systemic antibiotics (Wang et al., 2026).

Furthermore, in the realm of biological signaling, there is a paradigm shift from direct cell therapies toward cell-free and genetic approaches. While the utilization of bone marrow mesenchymal stem cells (BMSCs) has demonstrated potential (Xia et al., 2023), cell-based therapies face significant hurdles regarding *in vitro* expansion, immune rejection, and strict regulatory approvals. Consequently, exosome-mediated therapy is emerging as a highly promising alternative. Stem cell-derived exosomes—nanoscale extracellular vesicles rich in osteoinductive microRNAs and proteins—can stimulate endogenous cell migration and angiogenesis while entirely bypassing the risks associated with live cell transplantation (Liu et al., 2025; Tajafrooz et al., 2025). Concurrently, localized gene delivery systems utilizing non-viral vectors are being investigated to transfect resident fibroblasts and osteoblasts with plasmid DNA (D'Mello et al., 2016; Goker et al., 2019). This approach temporarily turns local host cells into localized “bioreactors, “ ensuring the sustained, physiological release of morphogens like BMPs or anti-inflammatory cytokines over the prolonged tissue remodeling phase (Chu et al., 2024).

### 5.3 Factors influencing clinical efficacy

The clinical success rate of regenerative therapy is strictly constrained by multiple critical factors. A primary prerequisite for any regenerative success is the meticulous and complete eradication of the pathogenic biofilm from the implant surface (Renvert and Polyzois, 2015). Residual pathogenic bacteria and their virulence factors—such as the proteases and LPS secreted by *P. gingivalis*—can rapidly reignite the local inflammatory cascade. These residual contaminants not only induce persistent inflammation but also directly inhibit the proliferation and differentiation of osteogenic cells, while simultaneously upregulating osteoclastogenesis via the RANKL/OPG pathway (Anitua et al., 2023). Consequently, failure to strictly control the microbial load at the surgical site may compromise the structural integrity of the barrier membrane and lead to graft failure (Khoury et al., 2019). Therefore, clinical protocols must combine mechanical and chemical methods for comprehensive debridement. Secondly, stringent case selection is fundamental to treatment success (Monje et al., 2025). Cases with three-dimensional bone walls (such as three-wall or four-wall defects) demonstrate significantly more predictable regenerative outcomes compared to open bone defects (Solderer and Schmidlin, 2020). Thirdly, contouring the exposed rough implant surfaces above the bone crest effectively reduces plaque retention risk, which is crucial for preventing recurrence (Yerramsetti et al., 2025). Finally, long-term standardized plaque control by patients and regular professional maintenance are essential for sustaining regenerative outcomes and preventing disease recurrence.

## 6 Challenges and future prospects

Despite significant progress made in basic research and clinical techniques, the comprehensive management of peri-implantitis still faces many challenges and also holds promise for future development directions.

### 6.1 Existing bottlenecks

The field currently faces multiple challenges. First, although various highly sensitive biomarkers have been identified, most remain at the laboratory research stage. Advancing detection technologies toward cost-effective, user-friendly POCT and achieving widespread adoption in primary healthcare institutions are critical issues that require urgent resolution. Second, the long-term efficacy stability and predictability of regenerative therapies still need improvement. In real-world clinical implementation, regenerative outcomes are heavily compromised by anatomical constraints, high technique sensitivity, and the inherent impossibility of absolute implant surface sterilization (Cho et al., 2025). Furthermore, substantial contradictions exist across clinical trials regarding the “gold standard” decontamination protocol, leaving clinicians without a universally reliable, evidence-based treatment algorithm (Khoury et al., 2019). Achieving functional osseointegration rather than mere bone defect filling, along with ensuring the long-term stability of regenerated tissues, remains an internationally recognized technical challenge. Furthermore, with the rapid development of multi-omics technologies, effectively integrating vast amounts of microbiomics, proteomics, and other data to extract clinically actionable insights and translate them into practical decision-making tools for clinicians requires deep collaboration between bioinformatics experts and clinical practitioners.

### 6.2 Research directions and development trends

Future research will focus on precision, minimally invasive approaches, intelligent technologies, and preventive strategies. The development of integrated diagnostic tools aims to consolidate multiple detection techniques, such as microfluidic chip-based platforms capable of simultaneously analyzing key pathogen DNA, aMMP-8 activity, and the RANKL/OPG ratio in PICF, enabling rapid multidimensional assessment of disease status. Personalized minimally invasive treatment strategies should be developed based on the patient’s microbiome and host response characteristics, such as targeted antimicrobial therapy using narrow-spectrum antibiotics or bacteriophages, combined with endoscopic visualization techniques to achieve precise debridement of deep lesions. The development directions of intelligent biomaterials and regenerative technologies include constructing responsive materials with dual functions of antibacterial and osteogenic properties, such as 3D-printed customized scaffolds capable of controlled release of active molecules in response to local microenvironmental changes, as well as exploring exosome-mediated cell-free regenerative therapies and local gene delivery technologies. The ultimate goal is to establish a comprehensive management system covering the entire life cycle of implants. Through the multi-stage coordination of high-risk screening, early warning, precise intervention, and regenerative repair, the system aims to achieve holistic management from disease prevention to functional reconstruction. This process requires deep interdisciplinary integration among dentistry, microbiology, immunology, biomaterials, and information science.

## 7 Conclusion

**Figure 4:**
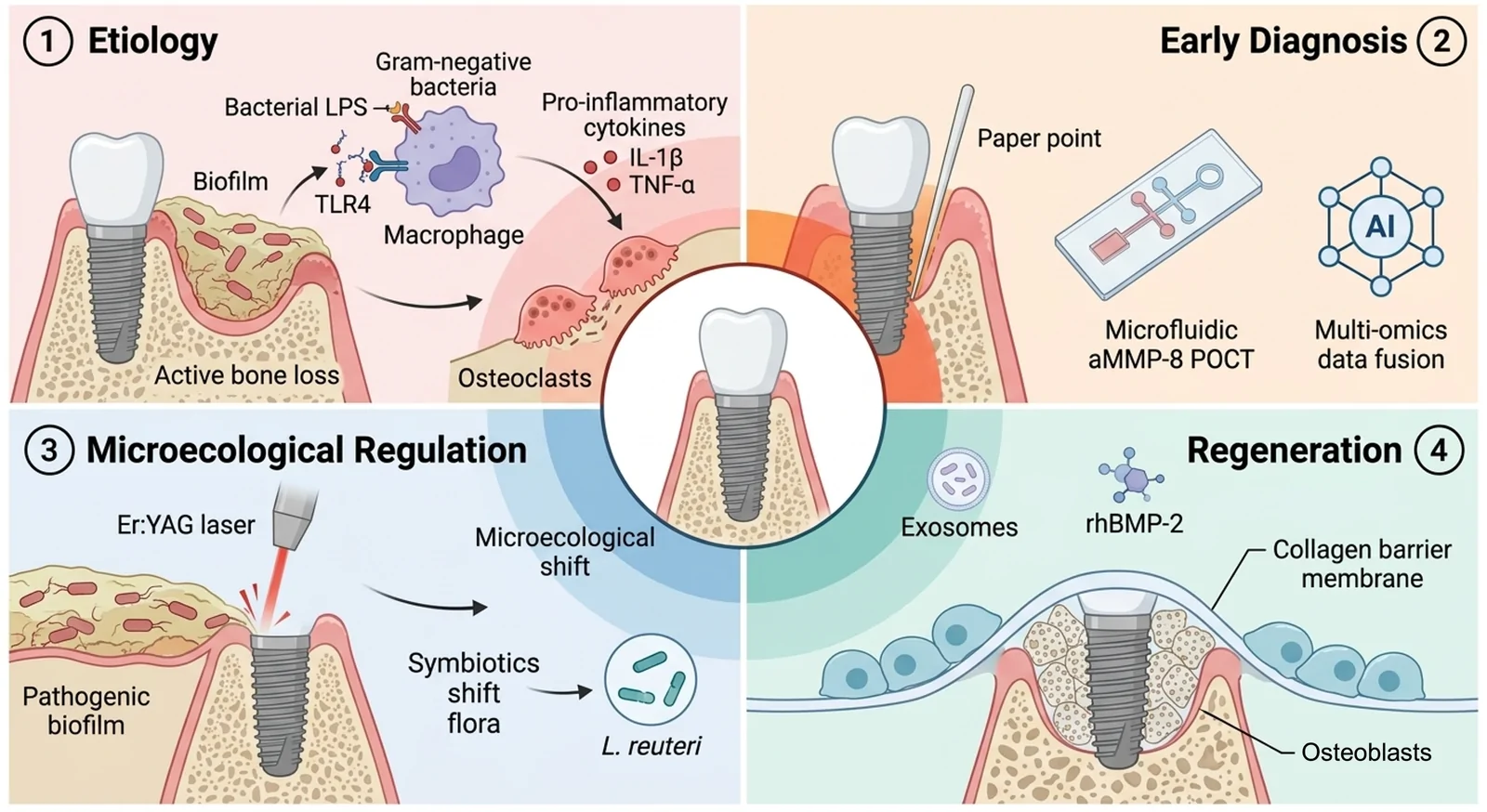
Integrated schematic of the four pillars in peri-implantitis management. This infographic summarizes the holistic framework for peri-implantitis management across four interconnected dimensions. Etiology: Pathogenic biofilm, dominated by Gram-negative anaerobes, drives host immune activation via bacterial LPS/TLR4 signaling, leading to osteoclast-mediated bone resorption. Early Diagnosis: Paper point sampling of peri-implant crevicular fluid (PICF), combined with microfluidic-based point-of-care testing (POCT) for biomarkers such as aMMP-8, and integrated with multi-omics data fusion and artificial intelligence, enables early and accurate disease detection. Microecological Regulation: Therapeutic interventions including Er:YAG laser debridement and probiotic supplementation (e.g., Lactobacillus reuteri) promote a symbiotic microbial shift, restoring microecological balance. Regeneration: Advanced regenerative approaches utilizing exosome-mediated therapy, recombinant human bone morphogenetic protein-2 (rhBMP-2), and resorbable collagen barrier membranes facilitate osteoblast-mediated new bone formation and functional osseointegration.

The holistic management of peri-implantitis integrates four critical dimensions—etiological elucidation, early diagnosis, microecological regulation, and regenerative therapy—as conceptually illustrated in **Figure 4**. The research paradigm for peri-implantitis has evolved from the traditional infection model to a dysbiosis-based host-microbe interaction disorder model. This shift in understanding has not only revealed the more complex pathological mechanisms of the disease but has also driven the development of early precision diagnostic technologies centered on molecular biomarkers. In clinical practice, it is essential to move beyond the singular approach of infection control and develop integrated treatment strategies that combine microbial modulation with tissue regeneration. The former aims to precisely eliminate pathogenic factors and restore microbial homeostasis, while the latter focuses on achieving predictable reconstruction of damaged tissue structures. Only through interdisciplinary integration and the translation of basic research findings into clinical diagnostic tools, therapeutic approaches, and biomaterials can the challenges of peri-implantitis be effectively addressed, thereby ensuring the long-term health and functional stability of oral implant rehabilitation.
